# Spontaneous cervical artery dissection: a fluoroquinolone induced connective tissue disorder?

**DOI:** 10.1186/s12998-018-0193-z

**Published:** 2018-07-09

**Authors:** James S. Demetrious

**Affiliations:** Wilmington, USA

**Keywords:** Cervical artery dissection, Fluoroquinolone, Carotid, Vertebral, Stroke, Infarct, Connective tissue, Collagen, Aneurysm

## Abstract

**Background:**

Spontaneous cervical artery dissections more often manifest in young people and have been associated with catastrophic consequences. Some indeterminate risk factors have been identified, making the diagnosis of developing dissections quite difficult. Fluoroquinolone antibiotics have been recognized for their degradative effects on connective tissue. Recent studies have implicated fluoroquinolones in the genesis of aortic artery aneurysms. It is the purpose of this paper to provide reasoning for a testable hypothesis of whether fluoroquinolones constitute a risk factor associated with cervical artery dissections.

**Methods:**

A PubMed search was conducted to investigate whether cervical artery dissection has been associated with fluoroquinolone use. An assessment of risk factors was made of hereditary connective tissue disorders, infection, and seasonal predisposition related to cervical artery dissection. These factors were considered in conjunction with reports of connective tissue toxicity associated with fluoroquinolone medications.

**Results:**

It appears that no reported cases of cervical artery dissection have previously been correlated with fluoroquinolone use. Heritable connective tissue disorders, infection, seasonal predisposition and condition latencies are associated with fluoroquinolone medications. Several recent articles have implicated fluoroquinolones with aortic dissections and aneurysm.

**Conclusion:**

A causal relationship of fluoroquinolone antibiotics to cervical artery dissection is plausible. The suppositions developed in this paper are insufficient to suggest that fluoroquinolones currently represent an established risk factor in the development of cervical artery dissections. Fluoroquinolones may indeed be a novel and previously unrecognized cause of cervical artery dissections.

## Introduction

The primary objective of this paper is to provide a testable hypothesis whether fluoroquinolone antibiotics constitute a risk factor associated with spontaneous cervical artery dissections.

### Epidemiology

Spontaneous cervical artery dissections are a common cause of stroke in young adults, with a mean age of occurrence of approximately 45 years and a slight gender predisposition favoring males 48–55% [1, 2]. During a 17-year study period, Lee et al. reported a mean annual incident rate of 2.6 to 3.1 per 100,000 population [3]. A seasonal variation has been reported with cervical artery dissection more likely to occur in the winter [4].

### Congenital risk factors

Heritable disorders such as Ehlers-Danlos syndrome, Marfan syndrome, osteogenesis imperfecta type 1, autosomal dominant polycystic kidney disease, and fibromuscular dysplasia have been reported as risk factors for cervical artery dissection [2]. While only 1–5% of patients with these connective tissue disorders have been identified with spontaneous vertebral artery dissection, 20% of patients have a yet unnamed connective tissue disorder [5].

### Environmental risk factors

A history of recent infection has been associated as a risk factor for cervical artery dissection [6, 7]. Extrapolating from a national database of outpatient antibiotic prescriptions in the United States, Suda et al. reported an average of 24.5% more antibiotic prescriptions were dispensed in the winter months than in the summer, over a 5-year study period with a mean outpatient consumption of quinolone antibiotics of 13.66% [8]. These combined factors may partially explain the seasonal variation and increased risk of dissection during winter months.

### Fluoroquinolone adverse effects

Often prescribed for urinary, respiratory, dermatologic and other infections, severe adverse effects of fluoroquinolones have been reported in the U.S., causing the Food and Drug Administration to communicate advisories related to safety and utilization:

- October 2008- Warning on tendon injuries with fluoroquinolone antibiotics.
- August 2013- Warning on peripheral neuropathy injuries with fluoroquinolone antibiotics.
- May 12, 2016- FDA approves safety labeling changes for fluoroquinolones.
- July 26, 2016- FDA Drug Safety Communication: FDA updates warnings for oral and injectable fluoroquinolone antibiotics due to disabling side effects.

Currently, the FDA has advised that fluoroquinolones should be reserved for use in patients who have no other treatment options for acute bacterial sinusitis, acute bacterial exacerbation of chronic bronchitis, and uncomplicated urinary tract infections because the risk of severe side effects outweighs the benefits in these patients. For some serious bacterial infections, the benefits of fluoroquinolones outweigh the risks, and it is appropriate for them to remain available as a therapeutic option [9].

### Fluoroquinolones and connective tissue toxicity

The FDA issued a Black Box warning for adverse effects associated with fluoroquinolones following surveillance data that associated these medications with tendinitis and tendon rupture. Wise et al. reported that the use of fluoroquinolones was associated with a 4-fold increase risk of Achilles tendinopathy and a 2-fold increase of tendon rupture in a database study of 6.4 million patients [10].

Collagen degradation due to fluoroquinolones reportedly involves the upregulation of matrix metalloproteinases resulting in a reduction in the quantity and quality of collagen fibrils [11]. A considerable latency from the commencement of fluoroquinolones to the onset of symptoms have been attributed to delayed mitochondrial toxicity, depletion, mutation, and cytotoxicity providing a foundation for reported occurrence of associated adverse effects [12].

In a review of 98 case reports of fluoroquinolone-associated tendinopathy, symptoms were reported as occurring within two hours of taking the medication to as long as six months after the cessation of treatment, with the median time of onset of six days [13]. The FDA reports that side effects occurred within hours to weeks after starting the fluoroquinolone, for an average of fourteen months to as long as nine years after stopping the medicines. Several cases reported that some side effects stopped or improved after discontinuation of the drug; others reported the side effects worsened or continued [9].

### Fluoroquinolones, aortic aneurysms, and cervical arterial dissections

Congenital disorders with collagen defects such as Ehlers-Danlos syndrome or Marfan syndrome have a predisposition to both aortic aneurysmal dilation and dissection [12]. Multiple studies suggest that exposure to fluoroquinolones have an associated increased risk of an aortic aneurysm and dissection [11, 14–16]. Collagen is a major extracellular matrix component of the aortic wall raising the concern that fluoroquinolones may cause or aggravate an aortic aneurysm and dissection. Lee et al. reported a 2-fold increase in the risk of an aortic aneurysm and dissection within 60 days of fluoroquinolone exposure [14].

The tunica adventitia of the aortic, carotid and vertebral arteries are comprised of dense irregular connective tissue containing loosely organized collagen fibers [17]. As fluoroquinolones may induce degradation of collagen causing aortic dissection and aneurysm, this raises the concern that fluoroquinolones may cause cervical artery dissections by a similar mechanism.

As previously reported by Schievink et al., of patients identified with spontaneous vertebral artery dissection, 20% of those patients have a yet unnamed connective tissue disorder [5]. A PubMed search using terms including: “fluoroquinolone”, “cervical”, “artery”, “dissection”, “vertebral”, and “carotid” was performed. No case reports, discussion or references were identified that associated cervical artery dissection and fluoroquinolone use.

## Discussion

It is plausible that fluoroquinolones may incite connective tissue degradation and play a contributory role in the genesis of cervical artery dissections. When considering the extent of adverse effects associated with fluoroquinolones, some multifactorial, interrelated risk factors and associations warrant further investigation regarding the etiology of spontaneous cervical artery dissection:

1. Connective tissue: Aortic and cervical arteries are comprised of connective tissue. Fluoroquinolone toxicity is associated with connective tissue degradation and aortic aneurysms. Adverse effects of fluoroquinolone medications may explain why otherwise normal; young populations experience cervical artery dissections.
2. Infections and seasonal relationships: Infections are associated with an increased risk of cervical artery dissection. The frequency of infections, fluoroquinolone utilization and cervical artery dissections increase during winter months. These temporal relationships may predispose patients to iatrogenic cervical artery dissections.
3. Latency: The latencies of clinical manifestations following the utilization of fluoroquinolone medications require careful consideration by clinicians.

When considering these factors, it is reasonable to ponder an associated causality between fluoroquinolone use and cervical artery dissection.

## Conclusion

A causal relationship of fluoroquinolone antibiotics to cervical artery dissection is plausible. Fluoroquinolones may indeed be a novel and previously unrecognized cause of cervical artery dissections. This hypothesis is insufficient to conclude that fluoroquinolones represent a current and established risk factor for the development of cervical artery dissections. An initial call for case reports and formal investigation is warranted. An extensive longitudinally observed population study might shed light upon this subject.
